# Challenges for providing health care in traumatized populations: barriers for PTSD treatments and the need for new developments

**DOI:** 10.1080/16549716.2017.1322399

**Published:** 2017-05-31

**Authors:** Evaldas Kazlauskas

**Affiliations:** ^a^ Department of Clinical and Organizational Psychology, Vilnius University, Vilnius, Lithuania

**Keywords:** Trauma, barriers, global mental health, PTSD

## Abstract

There is a growing recognition about the effects of traumatic experiences on mental health worldwide. With ongoing conflicts, natural disasters, interpersonal violence, and other traumatic events it is estimated that approximately 70% of the global population have been exposed to at least one lifetime traumatic experience. Research shows a substantial proportion of survivors, especially in low- and middle-income countries, would have a posttraumatic stress disorder (PTSD). During recent decades effective evidence-based treatments for PTSD have been developed. However, there are significant barriers to mental health services and trauma-informed treatments are not easily available for trauma survivors. From the perspective of social psychotraumatology several core barriers to trauma treatments were identified, including the lack of acknowledgment, and avoidance of disclosure. The need for cultural sensitivity in PTSD treatments, the potential of alternative ways of treatment delivery, and the involvement of non-professional volunteers are proposed as directions for future developments in the field.

## The burden of trauma and posttraumatic stress

Even though we wish our world was safe and secure, traumatic experiences are inevitable companions of human existence. Research suggests that the majority of a population are exposed to traumatic events in a lifetime, and that this is true worldwide. A recent epidemiological study revealed that even in high-income countries, such as the United States of America, 95% of the population experienced at least one lifetime traumatic event [1], and about 70% of Europeans are exposed to traumatic experiences [2].

Studies also demonstrate a remarkable resilience in coping with traumatic experiences. A majority of survivors successfully cope with traumatic experiences, utilizing inner resources, and social support from their social network. However, some survivors may have severe adjustment impairments after traumatic events. Since the inclusion of the Posttraumatic Stress Disorder (PTSD) in the Diagnostic and Statistical Manual of Disorders (DSM-III) in 1980, PTSD has now become one of the most common diagnoses among mental health professionals [3]. Still, we have rather limited knowledge on the prevalence of PTSD worldwide. Based on the epidemiological estimations, 12-month PTSD prevalence can range from 1% to 38% across different countries. Recent estimations indicate that the prevalence of PTSD over the last 12 months ranged from 1.1% to 2.9% in different age groups across Europe [4]. With a population of about half a billion, we can identify that at least 5 million have PTSD in Europe alone. And in a country in conflict, PTSD prevalence in the general population can be as high as 38% [5], indicating there are millions of survivors with PTSD globally.

This paper aims to discuss barriers for effective PTSD treatments, primarily in low- and middle-income countries, and identify several important future directions for reducing the burden of PTSD in traumatized populations.

## Effective treatments for PTSD

PTSD has a high cost for societies and there is worldwide demand for effective treatments of PTSD. Over the last few decades a number of evidence-based PTSD treatments have been developed [6]. However, these treatments have mostly been developed and tested in the high-income countries (USA, Canada, and Europe). Effective treatments, such as Trauma-Focused Cognitive Behavioral Therapy (TF-CBT) or Eye Movement Desensitization and Reprocessing (EMDR), although having strong evidence of efficacy have not been properly tested in a multicultural global context. The recently updated Cochrane review [7] of chronic PTSD treatments included 70 studies, of which only 7 studies (10%) originated from the setting of low- and middle-income Asian or African countries. Furthermore, even in European countries with developed health care systems there is considerable variety among countries in the availability of trauma-focused treatments [8].

## Barriers for effective trauma treatments

The treatment gap in mental health is worldwide [9]. Based on the recent findings from the traumatic stress research field, several major *barriers* for dissemination and delivery of effective PTSD treatments for traumatized populations important for health care policies were identified:

1. *Acknowledgment of survivors*. Across many cultures trauma survivors may experience stigmatization, and a lack of acknowledgment. The negative role of the lack of social acknowledgment has been demonstrated in several studies recently [10,11]. Negative attitudes towards trauma survivors in a society result in avoidant help-seeking behaviors. The lack of social acknowledgment of trauma and its effects results in inadequate health care policies on a national level [12].
2. *Avoidance and trauma disclosure*. One of the core PTSD symptoms is avoidance. Survivors with PTSD symptoms very often avoid disclosure of traumatic experiences, and trauma reminders, and may be reluctant to seek help. Studies on the disclosure of trauma have revealed that it is a significant predictor of PTSD symptoms [10,13,14]. In combination with a lack of acknowledgment of survivors, trauma disclosure may be affected. This results in avoidance of health care services, even in contexts where evidence-based treatments are available.
3. *Limited resources*. Evidence suggests that psychological treatments (TF-CBT, EMDR, etc.) for trauma survivors are the most effective treatments for PTSD [7]. However, qualified medical doctors or psychologists with training in trauma-focused treatments are needed to provide these treatments. Poor health care infrastructure, and the lack of training institutions, may be a significant barrier to providing trauma-informed evidence-based treatments in many countries. Surprisingly, even in many European countries access to high-quality evidence-based trauma-focused treatments is limited as reported by the recent analysis of trauma treatments in seven European countries [8].
4. *Ongoing conflicts and disasters*. It is extremely difficult to provide PTSD treatments in a country with an on-going conflict or even war. Findings from studies in post-conflict areas also indicate that the PTSD rates in these countries are significantly higher and could reach up to 40% in the general population [5].

## Future directions for the development of the field

Therefore, several future directions for the traumatic stress field of research which are relevant for national and international health care developments that could contribute to reducing the gap of untreated PTSD in traumatized populations are proposed:

1. *Cultural sensitivity*. We have substantial evidence of the cross-cultural validity of the PTSD diagnosis. However, there is a need for cultural sensitivity in PTSD treatment [15]. More cross-cultural studies are needed to validate the effectiveness of PTSD treatments in countries outside the high-income countries in North America and Europe. Moreover, therapists must be trained to be able to practice in multi-cultural settings in the context of globalization and migration.
2. *Alternative models of health care*. There are a growing number of studies providing evidence that Internet-based interventions can be similarly as effective as traditional face-to-face treatments [16]. Global initiatives of E-clinics development with online multi-language assessments and treatment modules could facilitate access to evidence-based treatments of PTSD. Self-help Internet-based programs for stress management could significantly reduce the cost of treatments, and might be a useful solution as a prevention and intervention method [17].
3. *Involvement of non-professional volunteers*. We will not have enough available trained professionals in evidence-based trauma treatments worldwide in the near future. We need to revise our concepts of mental health care delivery and utilize volunteers and other non-professionals who can disseminate effective self-help programs and assist in addressing the psychological needs of trauma survivors. Global train-the-trainers programs can facilitate training of volunteers and non-professionals to deliver treatments, possibly in self-help.

## Conclusions

The burden and the cost of traumatic events and PTSD are increasing. Only several barriers for trauma treatments were identified in this paper, and a few directions based on the current status of the traumatic stress field of research were proposed. Health care policy makers should be informed about the role of the social factors of PTSD, such as the importance of the social acknowledgment of survivors. However, the active role of international organizations, such as the World Health Organization (WHO), is also crucial for influencing health care policies on the regional and national levels. With the increasing body of evidence about the resilience of survivors, and the findings on the effectiveness of trauma-focused treatments, we could hope that positive changes are possible in the near future in overcoming barriers to treatment of trauma-related disorders.

## Paper context

This paper discusses the barriers for treatments of PTSD in traumatized populations and calls for action in this field. As the lack of resources and social contextual factors contribute largely to the PTSD treatment gap, several potential future directions in this field, such as the application of alternative e-health methods of treatment, volunteer involvement, and new attitudes toward survivors, are proposed.

## References

[1] Kilpatrick DG, Resnick HS, Milanak ME, et al National estimates of exposure to traumatic events and PTSD prevalence using DSM-IV and DSM-5 criteria. J Trauma Stress. 2013;26:537–3. 24151000 10.1002/jts.21848 PMC4096796

[2] Darves-Bornoz J-M, Jordi A, de Girolamo G, et al Main traumatic events in Europe: PTSD in the European study of the epidemiology of mental disorders survey. J Trauma Stress. 2008;21:455–462. 18956444 10.1002/jts.20357

[3] Maercker A, Brewin CR, Bryant RA, et al Diagnosis and classification of disorders specifically associated with stress: proposals for ICD-11. World Psychiatry. 2013;12:198–206. 24096776 10.1002/wps.20057 PMC3799241

[4] Wittchen HU, Jacobi F, Rehm J, et al The size and burden of mental disorders and other disorders of the brain in Europe 2010. Eur Neuropsychopharmacol. 2011;21:655–679. 21896369 10.1016/j.euroneuro.2011.07.018

[5] Steel Z, Chey T, Marnane C, et al Association of torture and other potentially traumatic events with mental health outcomes among populations exposed to mass conflict and displacement: a systematic review and meta-analysis. JAMA. 2009;302:537–549. 19654388 10.1001/jama.2009.1132

[6] Schnyder U, Ehlers A, Elbert T, et al Psychotherapies for PTSD: what do they have in common? Eur J Psychotraumatol. 2015;6:28186. 10.3402/ejpt.v6.28186 PMC4541077 26290178

[7] Bisson JI, Roberts NP, Andrew M, et al Psychological therapies for chronic post-traumatic stress disorder (PTSD) in adults. Cochrane Database Syst Rev. 2013;CD003388. 24338345 10.1002/14651858.CD003388.pub4 PMC6991463

[8] Kazlauskas E, Javakhishvilli J, Meewisse M, et al Trauma treatment across Europe: where do we stand now from a perspective of seven countries. Eur J Psychotraumatol. 2016;7:29450. 10.3402/ejpt.v7.29450 PMC4800285 26996534

[9] Kohn R, Saxena S, Levav I, et al The treatment gap in mental health care. Bull World Health Organ. 2004;82:858–866. 15640922 PMC2623050

[10] Mueller J, Moergeli H, Maercker A. Disclosure and social acknowledgement as predictors of recovery from posttraumatic stress: A longitudinal study in crime victims. Can J Psychiatry. 2008;53:160–168. 18441662 10.1177/070674370805300306

[11] Wagner B, Keller V, Knaevelsrud C, et al Social acknowledgement as a predictor of post-traumatic stress and complicated grief after witnessing assisted suicide. Int J Soc Psychiatry. 2012;58:381–385. 21596730 10.1177/0020764011400791

[12] Kazlauskas E, Zelviene P, Eimontas J “No posttraumatic stress disorder in Lithuania”: national health care fails to identify PTSD. J Trauma Stress. 2017;30:99–102. 28072906 10.1002/jts.22152

[13] Greenberg MA, Stone AA Emotional disclosure about traumas and its relation to health: effects of previous disclosure and trauma severity. J Pers Soc Psychol. 1992;63:75–84. 1494986 10.1037//0022-3514.63.1.75

[14] Frisina PG, Borod JC, Lepore SJ, et al Analysis of the effects of written emotional disclosure on the health outcomes of clinical populations. J Nerv Ment Dis. 2004;192:629–634. 15348980 10.1097/01.nmd.0000138317.30764.63

[15] Schnyder U, Bryant RA, Ehlers A, et al Culture-sensitive psychotraumatology. Eur J Psychotraumatol. 2016;7:31179. 10.3402/ejpt.v7.31179 PMC5055610 27473520

[16] Andersson G, Cuijpers P, Carlbring P, et al Guided Internet-based vs. face-to-face cognitive behavior therapy for psychiatric and somatic disorders: a systematic review and meta-analysis. World Psychiatry. 2014;13:288–295. 25273302 10.1002/wps.20151 PMC4219070

[17] Barak A, Grohol JM Current and future trends in internet-supported mental health interventions. J Technol Hum Serv. 2011;29:155–196.

